# Arterio-arterial malformation between a high origin radial artery and brachial artery within the cubital fossa - its clinical and embryological significance: a case report

**DOI:** 10.4076/1757-1626-2-6836

**Published:** 2009-07-24

**Authors:** Salvatore Docimo, Dellene E Kornitsky, Robert V Hill, David E Elkowitz

**Affiliations:** 1 Department of Surgery, Lutheran Medical CenterBrooklyn, NY 11220USA; 2 New York College of Osteopathic MedicineOld Westbury, NY 11568USA; 3 Department of Anatomy, New York College of Osteopathic MedicineOld Westbury, NY 11568USA; 4 Department of Biomedical Sciences, New York College of Osteopathic MedicineOld Westbury, NY 11568USA

## Abstract

**Introduction:**

Arterial variations of the upper extremity are commonly seen in the radial and ulnar arteries. Arterial variations can be damaged through iatrogenic means if not properly documented.

**Case presentation:**

A rare arterial anomaly was found in a 61-year-old female cadaver consisting of an arterio-arterial malformation between a high-origin radial artery and the brachial artery within the cubital fossa. The high-origin radial artery arose from the axillary artery, deep to the pectoralis minor muscle. It coursed superficially through the anterior compartment of the arm, converging with the more deeply placed brachial artery in the cubital fossa.

**Conclusion:**

Our finding demonstrates the still vast array of possible arterial varieties and the need for awareness in order to prevent iatrogenic injury. We also provide supportive evidence of intussusceptive angiogenesis’ involvement in the formation of larger vessels.

## Introduction

Arterial variations are common in the upper extremity [1] with the radial or ulnar arteries most frequently involved [2]. Typically, the radial and ulnar arteries arise from the bifurcation of the brachial artery within or just distal to the cubital fossa.

The presence of a high-origin radial artery is one of the more frequent arterial variations of the upper-extremity [3-4]. A high-origin radial artery, with an incidence of 14.27% in cadaveric samples and 9.75% in angiographic examinations, is commonly caused by an early bifurcation of either the axillary or brachial artery [5]. A high-origin of the radial artery is five times more likely to occur from the brachial artery than the axillary artery [1].

The present case describes a high-origin radial artery which briefly joined the brachial artery just proximal to the cubital fossa before bifurcating once more into the radial and ulnar arteries which then proceeded on their typical courses. The point where the high-origin radial artery and brachial artery briefly joined was a patent vessel.

Microscopic examination of the patent vessel demonstrated an embryological origin as no scar tissue was noted which could have suggested previous injury leading to a fistula formation. The malformation provides supportive evidence of intussusceptive angiogenesis which occurs by internal division of preexisting capillary beds [6] by protrusion of capillary endothelial cells into the lumen creating two new vessels from one vessel [7]. Intussusceptive angiogenesis has previously only been applied to capillary angiogenesis. However, we present what we believe to be the first gross and microscopic analysis of intussusceptive angiogenesis occurring in larger vessels. Furthermore, an appreciation and awareness of such arterial variations of the upper extremity is vital to preventing iatrogenic injury during clinical procedures.

## Case presentation

### Materials and methods

During the anatomical dissection of a 61-year-old Caucasian female human cadaver, we discovered a right, unilateral arterio-arterial malformation between a high-origin radial artery and brachial artery. We performed a thorough dissection and digitally photographed the arteries in relation to the normal nerves in the arm. The malformation and regions just proximal and distal to it were removed and placed in 10% buffered formalin for 24 hours followed by treatment with 70-100% ethanol and xylene. The specimens were embedded in paraffin and serially sectioned from proximal to distal at a thickness of 5 µm using a microtome. Sections were stained with hematoxylin and eosin and observed with an Olympus BX41 multihead microscope and digitally photographed.

### Results

**Figure 1.**
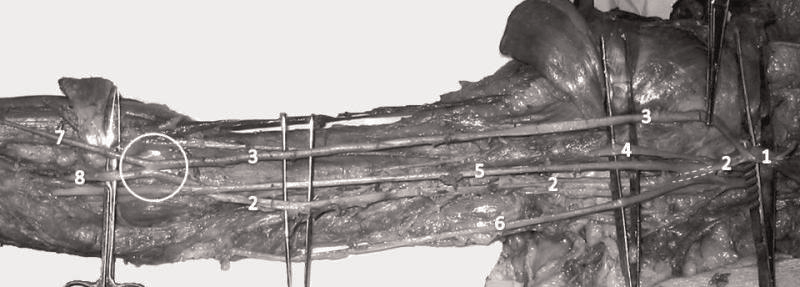
Gross dissection of the right shoulder, upper arm, and cubital fossa. **1**-Axillary Artery; **2**-Ulnar Artery; **3**-High-Origin Radial Artery; **4**-Musculocutaneous Nerve; **5**-Median Nerve; **6**-Ulnar Nerve; **7**-Radial Artery; **8**-Ulnar Artery; Circle-Arterio-Arterial Malformation.

**Figure 2.**
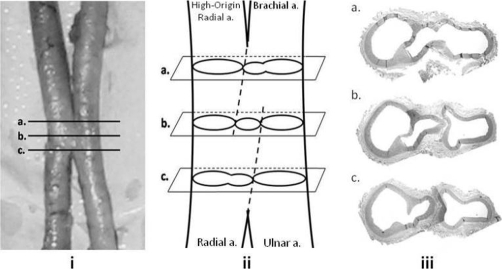
Gross sample of the arterio-arterial malformation **(i)** with corresponding levels **(a-c)** demonstrated in schematic form **(ii)** and in histological sections **(iii)**.

Prior to dissection, no scarring from previous injuries or surgical incisions was noted on the right arm. Figure 1 demonstrates the gross anatomy of our dissection. The radial artery originated from the axillary artery, as its origin occurred proximal to the insertion of the teres major muscle. Just distal to the pectoralis minor muscle, the axillary artery bifurcated into two equal sized arteries, one coursing deep and the other superficial to the median nerve. We considered these two arteries to represent a high-origin radial artery and the brachial artery. The brachial artery descended in the medial bicipital groove immediately lateral to the radial nerve. The high-origin radial artery descended in the anterior compartment of the arm and gave muscular branches to the biceps brachii. The high-origin radial artery then merged with the brachial artery at the cubital fossa before dividing again to give rise to radial and ulnar arteries distally. The radial and ulnar arteries then continued on their usual paths with no variations noted. Figure 2.i. demonstrates the gross sample of the arterio-arterial fistula.

The histological analysis of the arteries demonstrated that the juncture between the high-origin radial artery and brachial artery is patent. Figure 2.ii provides a schematic of the arterio-arterial malformation. As shown in Figure 2.iii.a, proximal to the malformation, the arteries exhibit two lumina corresponding to the high-origin radial artery and brachial artery. As the vessel progresses distally, the lumen of the brachial artery is portioned into two lumina giving rise to three lumina (Figure 2.iii.b). Figure 2.iii.b demonstrates the three lumina: the lumen on the left is formed by the high-origin radial artery, the lumen on the right belongs to the brachial artery, and the central lumen is formed by the malformation connecting the radial artery and brachial artery. Finally, the central lumen merged with the lumen of the high-origin radial artery to yield two lumina, the lumen on the left belonging to the radial artery and lumen on the right belonging to the ulnar artery (Figure 2.iii.c).

### Comment

Variations of the arterial anatomy of the upper extremity most commonly involves the radial artery [1,2]. Our discovery is important as it provides further insight into the embryological development of upper extremity arterial system. New vessel formation occurs via two processes: vasculogenesis and angiogenesis. Vasculogenesis, which occurs during embryonic development, is the process in which cells of the splanchnic mesoderm differentiate into endothelial precursor cells that eventually coalesce to form the initial primitive circulatory system of the embryo [6,7]. Angiogenesis is the expansion and remodeling of the vascular system using endothelial cells and vessels created during vasculogenesis [7].

Vasculogenesis occurs on day 18 of human development as cells of the splanchnic mesoderm differentiate into endothelial precursor cells which differentiate into endothelial cells [7]. Coalesence of these endothelial cells forms vesicular structures which join together to form vessels and a rough configuration of the embryonic circulatory system [7]. Our findings provide an insight into the process of angiogenesis rather than vasculogenesis. Angiogenesis, which occurs via sprouting or intussusception, is the expansion of the vascular system through existing endothelial cells which were originally generated by vasculogenesis [7]. Sprouting is a slow process that is able to bridge vascular gaps, such as during wound healing, and requires cell proliferation [8]. Whereas, intussusceptive angiogenesis can occur quickly within hours or minutes, can expand any existing capillary network, and does not rely on cell proliferation [8].

**Figure 3.**
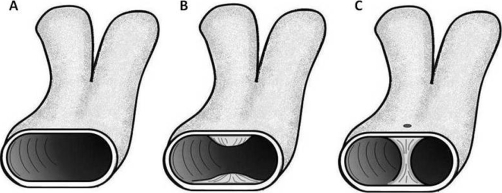
Four phases of intussusceptive angiogenesis. Phase 1 begins with the protrusion of the endothelial cells of the vessel wall into the lumen **(A-B)**. Formation of the pillar between the vessel wall encompasses Phase 2 **(C)**. Phase 3 **(C)** involves the perforation and invasion of the pillar by pericytes and myofibroblasts. Phase 4 involves all the structures of phase 3 plus collagen fibril formation.

Intussusceptive angiogenesis was first introduced nearly two decades ago as an alternative to the sprouting theory but is now believed to play a primary role in embryonic vascular development, further growth of existing capillary networks, and the formation of larger vessels [6]. Burri et al. [8] described intussusceptive angiogenesis as consisting of four phases. Phase 1 involves the protruding of the vessel walls into their lumen (Figure 3a,3b). Phase 2 involves the formation of the pillar between the endothelial protrusions (Figure 3c). Phase 3 involves the structures of Phase 2 with invasion of the pillar core by myofibroblasts and pericytes. Phase 4 involves all the structures of phase 3 plus collagen fibrils. Multiple pillars then forming parallel rows in capillary beds and fuse to form new capillary arteries.

Though the exact mechanisms of intussuceptive angiogenesis are poorly understood, its involvement in embryonic blood vessel formation has been well documented [9]. Intussusception and its method of capillary network expansion via pillar formation has been demonstrated in many organ systems such as the retina [6,10], 2000, 2002), the kidney [10], the human endometrium [11,12], in heart development [13], and cerebral vascularization [14]. The involvement of intussusceptive angiogenesis in large vessel formation is a new concept, however, having been only recently postulated by Djonov and colleagues [6,10]. Djonov et al. [10] demonstrated intussusceptive angiogenesis’ role in large diameter vessel formation by its involvement at the bifurcation points of vessels during development. Our finding provides supportive evidence of intussusceptive angiogenesis’ involvement in the formation of larger vessels as well.

**Figure 4.**
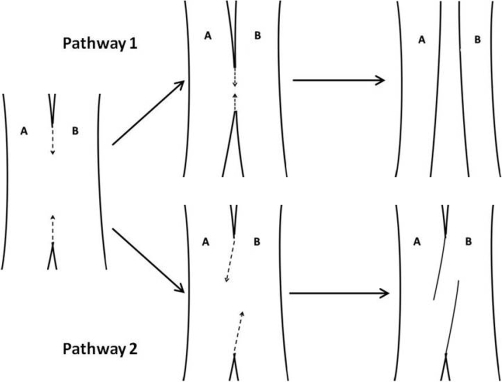
Pathway 1 depicts the possible correct process of intussusceptive angiogenesis occurring in large vessels during embryological development. As the pillars form and coalesce at the bifurcation points, the vessel splits from one vessel into two new vessels **(A, B)**. Pathway 2 depicts the failed process of intussusceptive angiogenesis that may have caused the arterio-arterial malformation. Rather than the distal and proximal bifurcations meeting and separating arteries **A** from **B**, the bifurcation points moved in opposite directions which may have caused the intussusceptive angiogenesis process to be halted.

The arterio-arterial malformation closely resembles a vessel in the mid-stages of intussusceptive angiogensis. Normally, the invaginating endothelial cells meet within the lumen to create a cylindrical tissue bridge wrapped by endothelial cells that extends across the vessel lumen [8]. Pericytes and interstitial fibroblasts invade the tissue bridge to cause an increase in its girth causing the vessel to split into two new vessels [7,8]. In our finding, it seems likely that the process was halted during the formation of the cylindrical tissue bridges or pillars. In Figure 4, Pathway 1 depicts the possible correct process of intussusceptive angiogenesis occurring in large vessels during embryological development. As the pillars of intussusceptive angiogenesis form at the bifurcation points, the pillars will increase in size and coalesces to cause vessels A and B to split apart into two new vessels. Pathway 2 depicts the failed process of intussusceptive angiogenesis that may have caused the arterio-arterial malformation. Rather than the distal and proximal bifurcations meeting and separating arteries A from B, the process of pillar formation and coalescence may have moved in opposite directions causing the intussusceptive angiogenesis process to be halted.

The malformation can be postulated to be caused by a dysfunction in the regulatory process of vessel formation. Vascular endothelial growth factor (VEGF), tyrosine kinase with immunoglobulin-like and EGF-like domains (Tie1, Tie2), and angiopoietin-1 (Ang1) have been proposed as regulatory factors in intussusceptive angiongenesis [7,9,15]. In mice lacking Tie2 and Ang1, they exhibited no initiation of vascular intussusceptive angiogenesis and death usually occurred in utero from abnormally large, leaky vessels [7]. Disruption of these regulatory factors may have played a role in the ceasation of intussusceptive angiogenesis in our cadaver. However, the exact mechanism remains unknown.

The clinical relevance of arterial malformations warrants accurate description and a keen understanding of the underlying causative mechanisms. The process of angiogenesis during tumor growth has often been an area targeted by drug treatments. Intussusceptive angiogenesis is known to play a role in tumor growth, such as colonic adenocarcinoma [15]. Additionally, intussusceptive angiogenesis has been described as the culprit in tumor recovery following irradiation and anti-angiogenic therapy. Using athymic nude mice injected with MMTV/c-neu murine carcinoma cells, Hlushchuk and colleagues [16] demonstrated a switch from sprouting to intussusceptive angiogenesis during tumor re-growth and recovery following treatments of irradiation and vascular endothelial growth factor-receptor tyrosine kinase inhibition. A thorough understanding of the underlying mechanisms governing intussusceptive angiogenesis may lead to improved treatments that counteract tumor angiogenesis.

An awareness of such arterial variations of the upper extremity may help prevent iatrogenic injury during clinical procedures. As the number of interventions utilizing the upper extremity arterial system increases annually, the need for understanding arterial anomalies is vital to prevent such complications such as thrombosis, gangrene, and even possible amputation [1]. Transradial percutaneous coronary intervention has experienced an increased growth in recent years due to the significant risks of femoral artery access [17,18]. Transulnar access for percutaneous coronary intervention procedures is becoming increasingly studied because the anatomical size variations of the radial artery often make it unsuitable for use [17]. Because arteries can be punctured relatively easily during percutaneous coronary procedures [17], an awareness of the existence of such a fistula is crucial as it can easily be damaged during an intervention.

Arterial branches of the upper extremity have also been used for other clinical procedures such as coronary bypass, flaps in reconstructive surgery, dialysis for chronic renal failure, and in interventional radiology [1,19]. The radial artery has also become one of the preferred entry sites for right vertebral artery access in neuroangiography [20]. Considering that ischemia of the upper extremity accounts for approximately 4% of all vascular procedures, knowledge regarding any type of arterial variations is crucial [1].

## Conclusion

The ever-increasing use of the upper extremity arterial system for clinical purposes dictates a need for documentation of arterial variations in order to prevent iatrogenic damage in the clinical setting. Furthermore, the gross and histological analysis of our finding provides new insights into the still novel theory that suggests intussusceptive angiogenesis plays a role in not only small vessel formation but large vessel development as well. However, further research into the mechanism behind the arterio-arterial malformation is needed as it remains elusive.

## Abbreviations

Ang 1: Angiopoietin-1
Tie1 & Tie2: Tyrosine kinase with immunoglobulin-like and EGF-like domains
VEGF: Vascular endothelial growth factor
